# Ten simple rules for writing a paper about scientific software

**DOI:** 10.1371/journal.pcbi.1008390

**Published:** 2020-11-12

**Authors:** Joseph D. Romano, Jason H. Moore

**Affiliations:** 1 Institute for Biomedical Informatics, University of Pennsylvania, Philadelphia, Pennsylvania, United States of America; 2 Department of Epidemiology, Biostatistics, and Informatics, University of Pennsylvania, Philadelphia, Pennsylvania, United States of America; 3 Center of Excellence in Environmental Toxicology, University of Pennsylvania, Philadelphia, Pennsylvania, United States of America; Dassault Systemes BIOVIA, UNITED STATES

## Abstract

Papers describing software are an important part of computational fields of scientific research. These “software papers” are unique in a number of ways, and they require special consideration to improve their impact on the scientific community and their efficacy at conveying important information. Here, we discuss 10 specific rules for writing software papers, covering some of the different scenarios and publication types that might be encountered, and important questions from which all computational researchers would benefit by asking along the way.

## Author summary

Computational researchers have a responsibility to ensure that the software they write stands up to the same scientific scrutiny as traditional research studies. These 10 simple rules make doing so easier by enhancing usability, reproducibility, transparency, and other crucial characteristics that aren’t taught in most computer science or research methods curricula.

## Introduction

In recent decades, scientific software has become a critical feature of virtually all research workflows [1]. Computational researchers and informaticians, therefore, have a responsibility to release and disseminate software in the same scientifically rigorous manner as other research protocols, datasets, and empirical studies released into the scientific community. Writing (and publishing) a peer-reviewed paper about a newly developed scientific software is arguably one of the best ways to do this—“software papers” can reach a massive number of potential users (even acting as advertisement for the software), are a great way to show that the software stands up to scientific scrutiny, and allow users to easily reuse and cite the software in their own research.

However, software papers are fundamentally different from other “traditional” research articles. The process of designing and implementing software is different from designing and carrying out bench experiments, clinical studies, or raw data analyses [2]. There are also differences in the “final product” of the research: Software studies, obviously, yield a piece of software to be directly reused, whereas other study paradigms provide new protocols, specific findings, and follow-up questions or hypotheses.

There are basically 2 types of software papers: (1) stand-alone papers that solely describe the software, usually in a shorter format than an article written about a traditional research study; and (2) a (more traditional) article describing an original research question that includes development of a piece of new software as one of its critical components. Examples of the former include the original papers describing Biopython [3], scikit-learn [4], and SAMtools [5]. The latter includes the papers that introduced Gene Set Enrichment Analysis [6], the Connectivity Map tools [7], and VIPER [8]. Although these options produce 2 very different styles of paper, the 10 simple rules presented below largely apply to both of them.

## Rule 1: Read the other “Ten Simple Rules” papers on coding

In order to have a good software paper, you first need to have good software. All of the other rules for writing great scientific software apply here, especially those that are already covered in other “Ten Simple Rules” articles. All impactful scientific software should aim to be robust [9], well documented [10], easy to use [11], and maintained under version control [12]. The advantages of making your software open source (with transparent licensing terms) and hosted on public repositories are widely acknowledged [13] and should be practiced regularly, unless there is a compelling reason not to. Evaluations, use cases, and demonstrative examples should make use of high-quality data that is ideally already well characterized [14,15].

## Rule 2: Know the most appropriate publication venues and submission types

Journals and conference proceedings that focus on computational areas of research frequently have article types that are dedicated specifically to descriptions of new software or databases, and these can be a great venue for disseminating information quickly, concisely, and to an audience with an assumed level of technical proficiency. It’s good to think early and reconsider often when finding a specific journal or conference. Make sure to pay special attention to any nonstandard requirements that journals impose—some require evaluations to use real (i.e., nonsynthetic) data [16], others have special reporting or data/software deposition requirements [17], and you should always consider whether your software (and desired paper style) match the mission statement and/or goals of the journal or meeting. Discussions with mentors, collaborators, and other colleagues can be hugely beneficial in this context; their past successes and failures can end up saving you from submitting to an unsuitable journal (and all of the headaches that come with it). Some examples of popular submission types and journals for software papers include *Bioinformatics* (submission type: “original papers” or “application notes”), *Nucleic Acid Research’s* yearly “Database issue” and “Web Server issue,” and *PLOS Computational Biology’s* “software articles.”

## Rule 3: Publish for users, not developers

In spite of Rule 2, you should always consider submission to noncomputational venues. As computational researchers, we often work in highly interdisciplinary areas, writing software that makes research in other fields easier, more efficient, and more scientifically robust. Scientists working in these fields are often extremely interested in hearing about new software tools that will help them on a daily basis, but they may not frequently search computationally focused journals or conference proceedings. Especially if the software is meant to be easily accessible to bench scientists or other noncomputational stakeholders, describing your software in a domain-specific journal is an excellent way to reach a wider audience. Furthermore, a paper describing an innovative new software tool in one of these journals has a great chance of standing out in comparison to other articles, especially when the field has highlighted a need for new software approaches to long-standing challenges.

However, a few things need to be kept in mind, especially when publishing in a noncomputational journal or conference. If software papers are uncommon in your field, there may not be an ideally suitable publication/article type, and you may need to be creative in how you organize your presentation of the software and your evaluation of its performance. Specifically, think of how your software can address a particular limitation or research question of interest to the field, and show an example demonstrating that it can do so. This can become a primary focus of the paper, or it can be one of several shorter “case studies” that show off useful functionality. Think about the story you want to tell, and what your target audience would find the most useful. Similarly, reviewers might be unfamiliar with how to assess and critique software papers. When in doubt, it never hurts to contact a journal editor or program committee member for guidance—they might even be able to direct the article to a set of reviewers they know have the needed technical expertise. If the publication venue asks authors for reviewer suggestions, you should be able to come up with a similar set yourself. You should also keep your readers in mind: If most of your intended audience have limited computational experience, you should actively cut down on jargon and technical details. These details can be added as supplemental data, published separately as a nontraditional article (e.g., via Zenodo, F1000, or similar), or even be moved entirely to online documentation (see Rule 6).

Good illustrative examples of software papers published in noncomputational journals are plentiful. Many older software papers were published in domain-specific journals, since most of the interdisciplinary fields that eventually led to computationally focused journals were still emerging. This can be seen, for example, in computational phylogenetics and cladistics, a field that began as early as the 1970s [18,19]. More modern examples of highly impactful software papers in domain-specific journals are also plentiful, like those introducing PLINK [20] and Circos [21].

## Rule 4: Create a long-term software management plan

In academia, affiliations, funding sources, and technology infrastructures change frequently. Researchers therefore assume a level of responsibility for keeping the products of our research available to the rest of the scientific community when things do change. When you release a new piece of software or body of code, you should establish guidelines to help ensure its persistence—otherwise, your papers, and those of others that rely on the software, will be negatively impacted. These guidelines form what can be thought of as a “software management plan” [22,23]. To create one, it can be helpful to ask yourself and your coauthors the following questions:

- Who is responsible for maintaining the software in the future, should affiliations change? The first author on the paper, the lab’s principal investigator (PI), or someone else?
- What is the cost (if any) of keeping the software and any related resources—relevant databases, web apps, application programming interfaces (APIs), etc.—online? What is the funding source? How will it be funded if this source is exhausted?
- Who owns the intellectual property (IP) behind the software? This is often the institution or company that employs the paper’s PI, but it may be different, and it may affect how the software is maintained in the long term. Furthermore, it is crucial to know how ownership of the IP will affect licensing [24]. Generally, it’s good practice to adopt the most permissive license that doesn’t violate ownership or usage/privacy policies.
- Will updates and bug fixes be provided? If the updates are major, will follow-up papers be published (see Rule 9)? Are any regular maintenance activities necessary, and if so, who will perform them?
- What will happen to the software if data or other resources it relies upon are no longer available?
- When and how will you archive the software? Online code repositories (e.g., via GitHub) make doing so easy, and tools like Zenodo and FigShare let you tie permanent DOIs to specific archives (see Rule 5).

Generally, software management plans aren’t outlined in the actual body of software papers, but an idea of how the lifecycle of the software will be handled—along with general policies and strategies for maintaining the software—are often included in online code repositories, such as in “Contributing” guides or the software’s README (e.g., [25–27]). General tips and guides on developing software management plans are in ample supply online [22,23].

## Rule 5: Safeguard against “link rot”

As papers age, it’s unfortunately quite common for hyperlinks to permanently break—the resource they point to has moved, has been taken offline, or affected by some other internet-related issue. This is known as “link rot,” and it is not just contained to academic articles—link rot can affect blogs, social media posts, web pages, and other digital resources [28]. However, it is especially prevalent in scientific articles—a 2013 study by Hennessey and Ge found that the median uniform resource locator (URL) lifespan is 9.3 years, with some falling far shorter than that mark [29]. While blogs, README documents, and source code can be edited to point to new links, peer-reviewed papers are static—unless you issue a correction or erratum, the URL you use at the time of publication is the URL that will be in that paper permanently.

Several relatively easy steps can be taken to prevent link rot in papers about scientific software. Institutional affiliations and website structure can change (as mentioned in Rule 4), so it is best to host web apps, APIs, software descriptions, example code, and other digital resources either on a dedicated domain, an independently hosted lab website, or on a free web hosting resource (e.g., using GitHub Pages). However, be familiar with how the host handles persistent links. For example, links on GitHub Pages sites can break if a repository is transferred to a new owner. When digital resources do need to move to a new URL, you should make an effort to set up URL redirection from the old location to the new location, which can usually be arranged with web server administrators. You should also set up persistent versioned releases of software and assign separate DOIs to point to the current software release at the time of the paper’s publication. Zenodo (for software releases tagged on GitHub) and FigShare (for data files, scripts, and other digital resources) are free services that track permanently archived research materials and assign DOIs that basically “solve” link rot when used effectively. Also, having a well-documented system for assigning meaningful URLs to individual resources can help to diagnose the cause if links do break. For example, “http://<domain>/protein/BRCA1” is likely far better than “http://<domain>/540/65df7.php?id=18427,” both from a usability and a maintenance perspective.

## Rule 6: Make a clear distinction between code documentation and research results

Whenever software is intended for reuse, high-quality documentation is crucial. However, peer-reviewed papers are arguably not where documentation should be presented. The paper should describe the software (including the design process, technical details, and algorithmic innovations) and any accompanying analyses. Any time you include code in the paper, you’re making a commitment to support the syntax and semantics in the code. Since it’ll be permanently visible to scientific users, changes that break the example code will likely lead to confusion and potentially result in alienating the users. If it’s especially important to show usage examples or other instructions on how to use the software, and they occupy more than a small handful of sentences, they should either (1) be moved to an appendix or supplemental materials document; or (2) be placed in the code’s documentation. If you have dedicated documentation pages online, it’s a great idea to provide a link to those pages in the body of the paper. To ensure consistency, the documentation should also be version controlled, and the link in the paper should point to the version of the documentation that is current at the time of writing. As a side note, sample input/output and example code that support results presented in the paper can also be placed in the software’s version control system and even integrated into the software’s test suite as acceptance tests [30].

## Rule 7: Be current with modern tooling and best coding practices

Many of the choices you make in the development of your software itself can have a profound effect on the longevity and scientific impact of the paper that describes it. If the software solves an important unmet need, yet is challenging to install, written in an obsolete programming language, and filled with bugs, it probably will not attain widespread use. Similarly, the paper would stand a high likelihood of falling by the wayside, if it even passes peer review. Fortunately, a relatively small amount of advance planning during the early stages of development can help avoid this particular issue. We find that the following guidelines are helpful here:

- Use a well-maintained programming language that runs on most modern systems.
- Publish your software on at least 1 packaging index so that users can install it with a single command. Using a continuous integration (CI) service can automate this process and reduce the likelihood of human error [31].
- Similarly, if possible, distribute your software both as raw source code and as a packaged or compiled version.
- As mentioned before, provide detailed documentation and instructions for use.
- If possible, provide ways for users to contribute to future development, especially in terms of bug fixes and requested features.

Doing so is also important for more fundamentally pragmatic reasons: A good way to encourage widespread use of software is to make it easy to install and use and to give it a fresh, modern look. Although this is not directly related to the scientific quality of the software or the paper, dissemination of research and research tools is an important part of the scientific process and should always be given special thought.

## Rule 8: Maintain consistency between code, documentation, websites, and papers

By its very nature, any software paper needs to manage references to (and between) an ecosystem of digital resources describing the software, including websites, source code repositories, documentation, example code, blog articles, and other media types, all of which refer to the same piece of software. Make sure to maintain consistency across this ecosystem. Use the same spelling, punctuation, and capitalization in any names you make up for your software. If you create a logo for the software, use it in multiple places. Make heavy use of links between different sites and resources so readers can find what they are looking for quickly and easily. An easy trick for ensuring version consistency is to include version numbers directly in URLs, where appropriate. For example, documentation pages might be given the URL “http://<domain>/version1/doc.” If the software is part of a larger body of research that has produced other pieces software, it might be a good idea to establish a naming system to indicate the relationship, while ensuring that each can be easily referred to without ambiguity. Don’t force acronyms in your naming either—keep acronyms simple or avoid them entirely.

## Rule 9: Plan for follow-up publications and update the software accordingly

More often than not, software development does not end after its first major release—rather, developers add new features and respond to bugs or other performance issues. This definitely applies to scientific software, too, which stems largely from the fact that good research is usually iterative [13] and conducted in stages that are either planned from the outset or guided by the successes and failures of earlier steps. Writing several papers along the way is more than a ploy to inflate citation metrics or boost a curriculum vitae (CV); it is a demonstration of rigorously following the scientific process, and it allows you to rapidly disseminate new findings to the community.

However, it is also important to know when it’s appropriate to write a follow-up paper. Things like bug fixes or minor usability enhancements are better suited for blog posts, version release notes, or message board/issue tracker threads. Discuss whether a new update constitutes a new scientific advancement and if that advancement solves a need that your user base currently faces. Generally, we aim to publish a new paper for every new major feature that is associated with a specific outstanding research question. For example, an ongoing project within our research group is the development of the Tree-based Pipeline Optimization Tool (TPOT)—an automated machine learning tool that uses genetic programming to automatically find machine learning pipelines that perform well on a given dataset [32]. In addition to the original publication describing TPOT, we have written follow-up papers for several major additions to the software, including new ways to specify pipeline templates [33] and support for deep learning [34].

## Rule 10: Prioritize visibility and availability

There is a frustrating scenario that plays out often when performing computational research: You find a paper describing a piece of free, publicly available software that perfectly solves a problem you have been struggling with for several weeks. The paper is a bit old, but the methods seem elegant and robust. However, after finally tracking down a copy of the software, you find that there is no way to make it run on any modern operating system. You spend a few hours trying to track down its (apparently nonexistent) documentation, and eventually give up, deciding that it is either impossible to get the software running or that it will simply take less time to implement it yourself. Problems like these can’t be entirely avoided—software ages, programming languages eventually fall out of favor, and dependencies change in ways that you as a user cannot fully control. However, as a developer, you can take steps to effectively prolong your software’s life, and some of these steps can be implemented directly in the paper that describes the software.

First, redundancy does not hurt. If you have a main informational website for the software, include a link in the paper, as well as on the source repository, in the documentation, and on lab and institutional websites. Make use of social media to promote your work and encourage coauthors to do the same [35]. A popular metric for determining the social impact of an article is the Altmetric Attention Score [36], which uses not only citation count but also things like social media mentions, news coverage, and representation in popular science publications. “Badge icons” (sometimes known as “shields”) used on websites, code repositories, and package indexes let you provide rich links to different parts of your software ecosystem (including the paper itself via DOIs) that are dynamic, informationally dense, and visually appealing. Finally, both your software paper and associated media related to the software can be optimized for search engines, which can dramatically increase their scientific impact [37].

Many of these apply in reverse, too. Once your paper is published (and prior to that, if you release a preprint), your websites and code repositories should point back to the paper, using its DOI when possible. It’s also helpful to explicitly instruct users how to cite your work and provide preformatted citations in several popular styles and/or BibTEX/EndNote/etc. files that can be exported directly to citation management software.

## Conclusion

Software papers are an important component of the scientific research ecosystem, benefiting users in many domains and with widely varying levels of computational expertise. Furthermore, academic publications (and their citations) currently provide the primary means by which scientific software developers and maintainers gain recognition for their work (fortunately, efforts are currently under way to change this—for example, [38,39] show how code contributions can be used as directly citable scholarly works). Following these 10 simple rules will help to ensure your software papers are easy to use, scientifically rigorous, and resistant to future changes in technology.
